# The Mediterranean Diet and Cancer: What Do Human and Molecular Studies Have to Say about It?

**DOI:** 10.3390/nu11092155

**Published:** 2019-09-09

**Authors:** Álvaro Hernáez, Ramón Estruch

**Affiliations:** 1 August Pi i Sunyer Biomedical Research Institute (IDIBAPS), 08036 Barcelona, Spain; 2 CIBER of Pathophysiology of Obesity and Nutrition (CIBEROBN), Instituto de Salud Carlos III, 28029 Madrid, Spain; 3 Department of Internal Medicine, Hospital Clínic, University of Barcelona, 08036 Barcelona, Spain

**Keywords:** Mediterranean diet, cancer, meta-analysis, prospective human studies, molecular mechanisms

Mediterranean diet (MD) is a well-known healthy dietary pattern, linked to: (1) high intakes of olive oil as main the culinary fat, plant-based foods (fruits, vegetables, legumes, whole grains, tree nuts, and seeds), and fish; and (2) a moderate consumption of white meat, eggs, dairy products such as yogurt and cheese, and wine always with meals [1]. Its protective effects on cardiovascular disease have been broadly described [2]. In addition, its consumption has also been associated with a decreased risk of suffering other chronic diseases such as diabetes, neurodegenerative disease, and even cancer. As published by Schwingshackl et al. having a high MD adherence is linked to a lower risk of developing colorectal, breast, gastric, liver, head/neck, and prostate cancers, as well as to a reduced cancer mortality in observational studies [3]. When focusing specifically on prospective trials, this meta-analysis highlighted significant decreases in the risk of suffering colorectal and breast malignancies and in cancer mortality among subjects with high MD adherence, and found single cohort studies describing decreases in the incidence of liver, gallbladder, and biliary tract malignancies. In line with these findings, two randomized controlled trials have studied the effect of this dietary pattern on cancer incidence. Adherence to the traditional Mediterranean diet in the context of the PREDIMED Study demonstrated a decrease in the incidence of invasive breast cancer in older women [4], while following a Mediterranean-type diet was associated with a lower general cancer incidence among patients in secondary prevention of cardiovascular diseases [5].

Schwingshackl et al. highlighted in their systematic approach that the consumption of fruits, vegetables, whole grains, and low-to-moderate doses of alcohol is particularly associated with the MD anti-cancer effects [3]. In meta-analyses of prospective human studies, fruit intake has been associated with a decreased risk of suffering total [6], colorectal [7], breast [8], gastric [9], bladder [10], lung [11], and liver cancers [12], as well as with lower cancer mortality [13]. Vegetable consumption has been linked in these studies with decreased incidence of total [6], colorectal [7], bladder [14], and lung cancer [11], and also with reductions in cancer mortality [13]. Finally, whole grain intake has been related to decreases in the incidence of colorectal [15] and gastric cancer [16], as well as with reductions in cancer mortality [17]. The consumption of other fiber and antioxidant sources such as legumes have also been associated with reductions in the risk of suffering colorectal [18] and prostate cancer [19] in other meta-analyses of prospective human studies (and intake of overall dietary fiber is linked to reduced risk of colorectal [20], breast [21], and ovarian cancer [22], and to lower cancer mortality [23]). Regarding ethanol, as stated by Schwingshackl et al. the attribution of anti-cancer effects to its consumption seems controversial, considering that it is categorized by the International Agency of Research on Cancer as a Group 1 carcinogen for humans [24] and by the World Cancer Research Fund as a convincing carcinogen for mouth, pharynx, larynx, esophagus, stomach, liver, colorectal, and breast malignancies [25]. However, it cannot be forgotten that a low-to-moderate wine consumption contributes to higher MD adherence scores (linked to the previously described benefits) and that no dose-response effect of the toxicity of alcohol or wine intake can be inferred from their conclusions. Additionally, wine provides high doses of some bioactive compounds such as flavonoids (potentially responsible for some anti-cancer effects) [26] and the possible counterregulatory effects of the entire dietary matrix against the toxicity of ethanol on cancer has not been explored. Finally, other changes in food consumption related to the MD may also contribute to its anti-cancer effects. On the one hand, polyunsaturated fatty acids (PUFAs) coming from nuts or fish can moderate low-grade inflammation states and, therefore, decrease cancer risk. In meta-analyses of prospective human studies, nut intake has been associated with decreases in total [27] and colon cancer risk [28], and fish consumption has been linked to reductions in the risk of suffering colorectal [15], breast [29], and liver cancer [30]. On the other hand, the moderations in the intake of red and processed meats in MD (substituted for healthier options) could also contribute to the anticancer effect. As observed in meta-analyses of prospective human trials, red meat consumption is associated with increases in the risk of developing colorectal [31], lung [32], and pancreatic cancer [33], and processed meat intake is linked to incremented risk of suffering colorectal [34], breast [35], gastric [36], prostate [37], and pancreatic malignancies [33].

MD consists of a healthy nutrient matrix whose individual components may moderate cancer risk by complementary mechanisms (Scheme 1).

The first one is focused on the role of oxidative stress on cell proliferation, since MD is known to be an antioxidant-rich dietary pattern [1]. On the one hand, antioxidants may directly neutralize reactive species of oxygen and nitrogen (RONS). RONS are able to promote the activation of signaling pathways such as those related to phosphoinositol-3-kinase (PI3K) and some mitogen-activated protein kinases (MAPKs) [38]. PI3K- and MAPK-related signaling cascades are physiologically initiated by growth factors and cytokines in different cell types and promote cell proliferation (essential in body growth and defense). In addition, these pathways activate the nuclear factor kappa beta (NF-κβ), a protein complex responsible for the production of cytokines and growth factors, which can in turn induce a positive feedback in the previous signaling pathways [39]. The RONS-mediated stimulation of PI3K- and MAPK-related pathways out of physiological circumstances leads to an uncontrolled acceleration of cell cycle, division, and low-grade inflammation, and these processes may promote cancer development [38]. Therefore, the antioxidant-mediated neutralization of RONS may reduce cancer risk. On the other hand, RONS may also oxidize DNA nitrogenous bases (especially guanine and adenine), which are no longer read correctly, and induce the appearance of mutations. These mutations may increase cancer risk whether they silence genes that downregulate cell proliferation or increase the expression/function of proliferative genes [40]. Therefore, the neutralization of RONS may decrease the mutation frequency rate in DNA. In addition, other typical MD antioxidants such as flavonoids regulate the previous pathways beyond their capacity to scavenge RONS. First, flavonoids have shown to directly modulate the excessive activation of PI3K- and MAPK-related signaling pathways [41]. Second, some flavonoids promote the activation of AMP-activated protein kinase. It is an enzymatic complex able to decrease the synthesis of lipids and proteins, to moderate NF-κβ activation, to slow down cell cycle, and to promote the synthesis of DNA-repairing and antioxidant enzymes [42,43], all of them essential processes in cancer development. Finally, flavonoids have also shown to inhibit some cytochrome P450 subunits involved in the activation of pro-carcinogens into active carcinogens, as well as to induce some phase II enzymatic processes (glucuronidation, conjugation with glutathione) responsible for carcinogen metabolism. Therefore, they promote a lower activation and a greater elimination of these substances [44].

Fiber is also a key component in the MD healthy nutrient matrix because of its several mechanisms. First, dietary fiber (and the prebiotic one in particular) is metabolized by the intestinal microbiota in a process that leads to the release of short-chain fatty acids (SCFAs), essentially butyric, propionic, and acetic acids [45]. Locally, these SCFAs cannot be used as energy source by cancerous colonocytes, accumulate, and inhibit the action of histone deacetylase enzymes in these cells. This process alters the epigenetic regulation of gene expression in cancerous colonocytes, decreasing their proliferation and promoting their apoptosis [46]. SCFAs can also bind to specific receptors in some enterocytes (GPR family receptors such as GPR43 and GPR109), which lead to the downregulation of NF-κβ. The activation of these receptors decreases the local pro-inflammatory responses that can promote the development of malignant enterocytes [47]. Some local and circulating immune cells also present GPR receptors on their surface (lymphocytes, neutrophils, dendritic cells) and its regulatory function on carcinogenesis may also be boosted by SCFAs [47,48]. Second, dietary fiber in an essential nutrient for the proliferation of probiotic bacteria. Probiotic species do not release pro-inflammatory endotoxins (e.g., lipopolysaccharide, mainly released by dysbiotic, unhealthy populations), avoid the excessive growth of other non-beneficial bacterial strains, and contribute to maintaining a correct intestinal permeability. Thus, fiber decreases the generation and absorption of endotoxins, and is able to promote low-grade inflammation because of their capacity to stimulate immune cells such as macrophages [49,50]. Finally, dietary fiber also contributes to decreasing glycemic index of foods. Low glycemic index carbohydrates are associated with lower post-prandial RONS peaks [51], which will decrease RONS-mediated cancer promotion. In addition, low glycemic index carbohydrates are also related to lower levels of insulin and insulin-related growth factors (such as insulin-like growth factor-1), therefore decreasing the uncontrolled promotion of growth responses [52].

Other MD dietary characteristics may also contribute to its cancer prevention effects. On the one hand, its richness in unsaturated fats (monounsaturated fatty acids –MUFAs– and PUFAs) may contribute to decreasing low-grade inflammation. First, MUFAs and PUFAs are necessary activators for the biochemical response of peroxisome proliferator-activated receptors, known to be able to downregulate NF-κβ-related signaling pathways [53]. Second, PUFAs are able to bind to fatty acid receptors such as GPR120 in some immune cells (e.g., macrophages), decreasing their pro-inflammatory activation [54]. Finally, PUFAs are also transformed into less pro-inflammatory eicosanoids (prostaglandins, thromboxanes, and leukotrienes) when they are metabolized by cyclooxygenases and lipoxygenases [55]. On the other hand, the substitution of red and processed meats for other healthier options in MD can also contribute to its anti-cancer capacity. Several substrates originating in the large intestine by the bacterial metabolism on the digestion residuals of red and processed meats (hydrogen sulfide, other sulfur compounds, nitrosamines, ammonia, amino acid metabolism residues, etc.) may compromise the intestinal integrity. This process increases the colon permeability to pro-inflammatory endotoxins such as lipopolysaccharide, released by dysbiotic bacteria [56]. In addition, cooking derivatives of these foods and some components of processed meats (polycyclic aromatic hydrocarbons, heterocyclic amines, *N*-nitroso compounds) are pro-carcinogenic agents [57]. Polycyclic aromatic hydrocarbons are also known to promote pro-inflammatory responses through the activation of aryl hydrocarbon receptors [58].

In summary, MD seems clearly linked to prevent the development of cancer, as reported in observational studies and their meta-analyses. Its individual components (fruits, vegetables, whole grains, legumes, nuts, fish, and a reduced intake of red/processed meats) have also been linked to cancer prevention benefits in meta-analyses of prospective human studies, with several molecular mechanisms supporting this hypothesis. However, further efforts in the context of dietary intervention trials are needed to confirm this protective effect with the highest level of scientific evidence.

## Figures and Tables

**Scheme 1:**
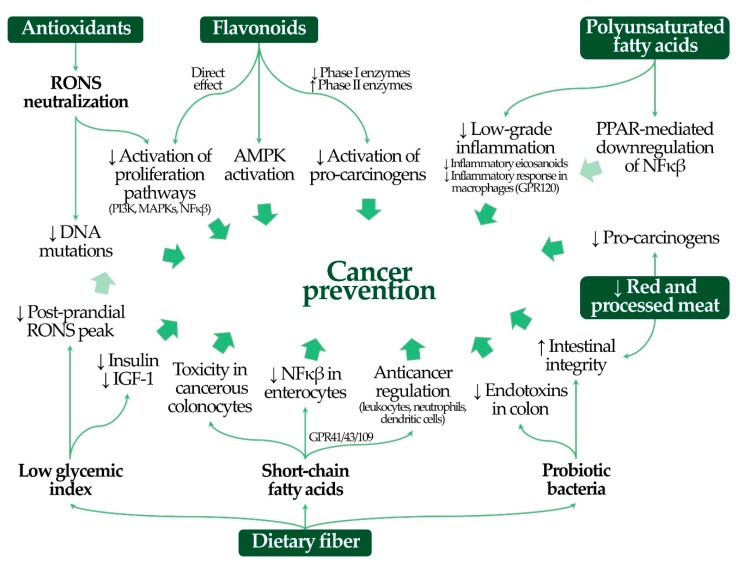
Molecular mechanisms of Mediterranean diet bioactive dietary components on cancer prevention. AMPK: AMP-mediated protein kinase; IGF-1: insulin-like growth factor-1; MAPKs: mitogen-activated protein kinases; NFκβ: nuclear factor kappa beta; PI3K: phosphoinositol-3-kinase; PPAR: peroxisome proliferator-activated receptor; RONS: reactive species of oxygen and nitrogen.
